# Declining Physical Performance Associates with Serum FasL, miR-21, and miR-146a in Aging Sprinters

**DOI:** 10.1155/2017/8468469

**Published:** 2017-01-03

**Authors:** Reeta Kangas, Timo Törmäkangas, Ari Heinonen, Markku Alen, Harri Suominen, Vuokko Kovanen, Eija K. Laakkonen, Marko T. Korhonen

**Affiliations:** ^1^Gerontology Research Center, Department of Health Sciences, University of Jyvaskyla, Jyvaskyla, Finland; ^2^Department of Medical Rehabilitation, Oulu University Hospital, Oulu, Finland; ^3^Center for Life Course Health Research, University of Oulu, Oulu, Finland

## Abstract

Aging is associated with systemic inflammation and cellular apoptosis accelerating physiological dysfunctions. Whether physically active way of life affects these associations is unclear. This study measured the levels of serum inflammatory and apoptotic molecules, their change over 10 years, and their associations with physical performance in sprint-trained male athletes. HsCRP, cell counts, HGB, FasL, miR-21, and miR-146a were measured cross-sectionally (*n* = 67, 18–90 yrs) and serum FasL, miR-21, and miR-146a and their aging-related associations with physical performance were assessed over a 10-year follow-up (*n* = 49, 50–90 yrs). The cross-sectional study showed positive age correlations for neutrophils and negative for lymphocytes, red blood cells, HGB, FasL, and miR-146a. During the 10-year follow-up, FasL decreased (*P* = 0.017) and miR-21 (*P* < 0.001) and miR-146a (*P* = 0.005) levels increased. When combining the molecule levels, aging, and physical performance, FasL associated with countermovement jump and bench press (*P* < 0.001), miR-21 and miR-146a with knee flexion (*P* = 0.023; *P* < 0.001), and bench press (*P* = 0.004; *P* < 0.001) and miR-146a with sprint performance (*P* < 0.001). The studied serum molecules changed in an age-dependent manner and were associated with declining physical performance. They have potential as biomarkers of aging-related processes influencing the development of physiological dysfunctions. Further research is needed focusing on the origins and targets of circulating microRNAs to clarify their function in various tissues with aging.

## 1. Introduction

Physical exercise affects inflammatory state. The common understanding is that an acute bout of exercise results in a temporal inflammatory response, while regular training has a protective anti-inflammatory effect [1–4]. In addition to immune cells, skeletal muscle tissue contributes to the inflammatory response by releasing inflammatory molecules, such as TNF-*α* and IL-6, following of acute exercise [5]. During inflammation, whether induced by age, disease, or acute exercise, controlling the cellular balance of the inflammatory cells is important. Apoptosis, or programmed cell destruction, is a crucial mechanism for maintaining cellular balance in all tissues. Disruptions in the cellular homeostasis lead to either an accumulation of poorly functioning cells or a deficit of important cells, both recognized in aging. One way to regulate immune cell homeostasis, and subsequent immune response, is through Fas ligand - Fas receptor (FasL-Fas) interaction on the cell surfaces, which leads to target cell destruction [6]. There are indications that habitual training influences the apoptotic processes of the immune system. According to Mooren et al. [7], the basal levels of leukocyte apoptosis as well as the levels of exercise-induced apoptosis are distinct between highly trained and poorly trained men, suggesting training-induced adaptation to leukocyte homeostasis.

Traces from inflammation and cellular apoptosis can be detected from the circulation by measuring specific molecules from the blood. In addition to the classical inflammation (e.g., CRP, IL6, and TNF-*α*) and apoptotic markers (cytochrome c, Fas, and FasL), the small RNA molecules called microRNAs (miRs) are novel and potentially even more sensitive tools for screening these physiological processes. miRs are noncoding RNAs regulating gene expression by blocking the translation of specific target mRNA into proteins. miRs are released, either actively or passively, from different cell types into the blood stream, where they reflect the gene regulation changes in their cells of origin. Their role in intercellular communication has also been recognized [8]. In healthy conditions, circulating miRs most likely originate from blood or epithelial cells or from organs with high vascularization, including skeletal muscle cells [9]. Owing to trauma, cancer, cardiovascular disease, or other conditions influencing metabolism, the miR signature in the circulation changes.

mir-21 and miR-146a are among the miRs that seem to be responsive to various physiological stimuli. These miRs are associated with aging-related processes such as senescence and inflammation, miR-21 having proinflammatory and miR-146a having anti-inflammatory effects [10, 11]. Both miRs induce apoptosis by targeting FasL-Fas signaling, which strengthens the interplay of these molecules with each other and their role as regulators of immune cell homeostasis [12, 13]. Circulating miR-21 has also been shown to promote cachexia-related apoptosis in skeletal muscle cells [14]. Physical exercise creates a whole body adaptive responses that also affect miR regulation and expression in different cell types and subsequently in the circulation [15–17]. miR-21 and miR-146a have also been shown to be highly responsive to physical exercise. The pioneering work by Baggish et al. [15] demonstrated that these miRs were associated with cardiovascular/musculoskeletal adaptations and low-grade inflammation and changed in response to acute exercise as well as sustained training. Circulating miR-146a levels were upregulated by acute cycling exercise before and after 90 days of sustained row training, whereas miR-21 was only upregulated by acute exercise prior to the sustained training period. In addition, the highest levels of miR-146a during exercise were found to correlate with peak VO_2max_ [15], whereas miR-21 levels have been shown to be upregulated in males with low VO_2_ max, indicating the contrasting roles of these two miRs [18]. Nielsen et al. [17] demonstrated that miR-146a decreased immediately after a bout of acute exercise, whereas the basal levels of miR-21 were downregulated after 12 weeks of endurance training. Both miR-21 and miR-146a have also been shown to exist at different levels in the plasma of young male endurance and strength trained athletes [19]. The above-mentioned studies demonstrate that these specific miRs detected in the circulation are affected differently by exercise type and duration and have potential as indicators of physiological changes [15, 16, 19, 20].

In order to evaluate if circulating FasL, miR-21, and miR-146a have the potential as biomarkers of training adaptations in aging athletes, longitudinal studies are needed. The aim of this study was to determine whether circulating FasL, miR-21, and miR-146a levels change over 10-year period among competitive male masters sprinters with a long-term training background and to assess their associations with physical performance measures and aging.

## 2. Methods

### 2.1. Study Design and Ethics

**Figure 1:**
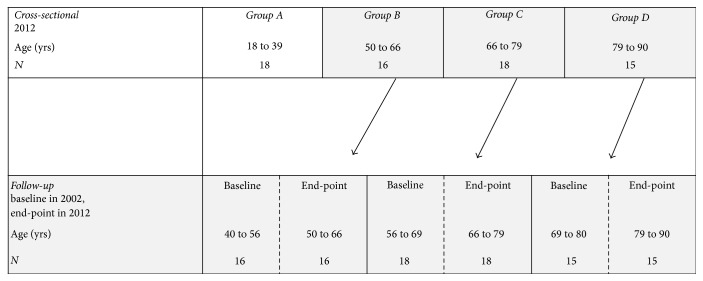
Descriptions of the study designs needed in the current study.

This study is part of an ongoing Athlete Aging Study (ATHLAS) [21–25] on young adult athletes and masters athletes from different sport disciplines. The participants were recruited from the memberships of Finnish athletic organizations. The present study comprised male sprinters. The sprinters had a long-term background in sprint training and had been successful in national or international 100–400 m sprinting events. Both cross-sectional and longitudinal study designs were used (Figure 1). In the cross-sectional analysis (using data from follow-up measurements conducted in 2012), the sprinters were divided into four age groups: (A) 18 to 39 years (*n* = 18), (B) 50 to 66 years (*n* = 16), (C) 66 to 79 years (*n* = 18), and (D) 79 to 90 years (*n* = 15). In addition, baseline measurements (conducted in 2002) were available for 49 older masters sprinters (aged 50 to 90 years at follow-up) belonging to age groups B, C, and D, thereby allowing longitudinal analysis. Both the baseline and the follow-up measurements consisted of similar standardized two-day measurements.

Prior to the measurements, training and health history were elicited and evaluated using questionnaires. The average number of training years among the masters athletes at baseline was 34.1 ± 15.1 years. More detailed self-reported training histories (training frequency, sprint-specific training hours, and other training hours) and their changes over 10 years are presented in Tables 1 and 3. The sprint-specific training hours per week included sprints, jumps, and strength training, while the other training hours included all other notably strenuous physical exercises. Subjects over age 55 underwent a medical examination. Current ability to participate in physically demanding measurement was assessed ad hoc, individually by the study physician. The health of all the participants was in general good with no acute conditions (infections, traumas) or functionally limiting chronic neurological, cardiovascular, endocrinological, or musculoskeletal conditions. In a few cases, some physical tests were not performed due to local musculoskeletal pain.

The study was conducted according to the guidelines of the Declaration of Helsinki. All participants were informed a priori about the possible risks and discomfort of the physical and clinical measurements. Written informed consent, including permission for the use of the gathered data for research purposes only, was provided by the study subjects. The study protocol was approved by the ethics committees of the University of Jyväskylä (in 2002 for baseline) and the Central Finland Healthcare District (in 2012 for follow-up).

### 2.2. Physical Performance and Body Composition Measurements

Participants were instructed to rest (no heavy training or competition) two days before the measurements. On the first measurement day, the participants performed a maximal 60 m sprint twice on an indoor running track with spiked running shoes. Explosive force production of the lower limbs was measured by a vertical countermovement jump (CMJ). Isometric knee flexion force and isometric upper limb force were measured by bench press performance using a David 200 dynamometer (David Fitness and Medical Ltd., Outokumpu, Finland). The best of 2 or 3 trials was recorded in the subsequent analyses. In addition, the assessment of total body fat and lean mass (LBM) was performed with bioelectrical impedance (Spectrum II, RJL System, Detroit, MI). More detailed descriptions of the measurements are given elsewhere [22–25]. Participants' meals during the measurement days were arranged by the research organizers.

### 2.3. Serum Analyses

To obtain serum, venous blood was collected on the second measurement day under standard fasting conditions at least 12 h after exercise. The serum samples were stored at −80°C until analyzed. Serum FasL concentration was measured using a Human Fas Ligand/TNFSF6 Quantikine® ELISA Kit (R&D Systems, Minneapolis, MN, USA) according to the manufacturer's protocol. Reactions were performed as duplicates and a common sample was added to each plate in order to observe whether controlling for interassay variation was necessary. HsCRP measurements at baseline and follow-up were performed with an Immulite 1000 Immunoassay System. Leukocyte counts were part of the standard medical complete blood count analysis.

### 2.4. RNA Extraction and miR Analyses

The RNA extraction methods and miR-21 and miR-146a analyses have been described previously [26]. Briefly, total RNA was isolated from 100 *µ*l of serum with a total RNA purification kit (Norgen Biotek Corporation, Thorold, ON, Canada) according to the manufacturer's protocol. Synthetic* C. elegans* Cel-miR-39 (5′-UCA CCG GGU GUA AAU CAG CUU G-3′, Invitrogen) (25 fM, concentration determined by dilution series) was added at the lysis step to all of the samples as a spike-in control in order to monitor the efficiency and uniformity of the RNA extraction and qPCR procedure. RNA was reverse-transcribed to cDNA (*V*_tot_ = 10 *μ*l) by a Taqman reverse transcription kit and the qPCR (*V*_tot_ = 10 *μ*l) was performed with a Taqman Universal Mastermix II NO Ung using Taqman miR assays (hsa-miR-21: 5′-UAGCUUAUCAGACUGAUGUUGA-3′, hsa-miR-146a: 5′-UGAGAACUGAAUUCCAUGGGUU-3′) (Device: Applied Biosystems, ABI 7300).

Ct values less than 35 were accepted for the analysis. All the samples were normalized to a reference sample of an average sprint athlete across the plates. ΔCt values were calculated as ΔCt = mean  Ct_miR-X_ − mean  Ct_miR (reference)_. Each reaction was performed in duplicate and the relative expressions were calculated by using the 2^−ΔCt^ method. To observe the possible contaminations and primer-dimers, no template controls (NTCs) were used in either the RT or qPCR reactions.

### 2.5. Statistical Analyses

In the age-correlation analyses of the cross-sectional design, Pearson's correlation coefficient was used for continuous variables and Spearman's correlation coefficient for ordinal variables. The paired sample *t*-test for parametric variables and Wilcoxon signed rank test for nonparametric variables were used in the longitudinal comparison in the 10-year follow-up design. In addition, Generalized Estimating Equations (GEE) models were constructed to study the association of FasL, miR-21, and miR-146a with the performance measures over age. The model was based on the two measurements points (2002, 2012) used in the longitudinal study with unstructured working correlation matrix specification and athletes' age as the descriptive metric of time. As the effect of age is generally nonlinear we used polynomial terms (quadratic and cubic) of age to include curvature in modeling the age-related effect of the FasL and the miRs on the outcomes. The results were adjusted for LBM. More detailed prediction equations based on GEE-models are presented in the supplementary data (S1 in Supplementary Material available online at https://doi.org/10.1155/2017/8468469).

## 3. Results

### 3.1. Participant Characteristics and Serum FasL and miR Levels in Different Age Groups

Participants' characteristics in the different age groups and age correlations of the variables are presented in Tables 1 and 2. The age correlations are presented in two different ways: (1) inclusive of all the participants (ages 18 to 90) and (2) inclusive only of the masters athletes (ages 50 to 90). A modest negative correlation was observed between age and training frequency when all the athletes were included in the analyses (*r* = −0.659, 95% CI = −0.8 to −0.5, *P* < 0.001), with age accounting for 43.3% of the variation in training frequency. The same correlation was very low when only the masters athletes were included (*r* = −0.176, 95% CI = −0.4 to 0.1, *P* = 0.241), with age accounting only for 3.1% of the variation. Among the physical performance measures, a high positive correlation was found between age and 60 m sprint time among all the athletes (*r* = 0.898, 95% CI = 0.8 to 0.9, *P* < 0.001), with age accounting for 80.6% of the variation in sprint time. The pattern was similar when only the masters athletes were included in the analyses (*r* = 0.841, 95% CI = 0.7 to 0.9, *P* < 0.001), with age accounting for 70.7% of the variation in sprint time. The other physical performance measures showed a modest to high negative correlation with age when all the athletes were included (CMJ: *r* = −0.81, 95% CI = −0.9 to −0.7, *P* < 0.001, 65.6%; knee flexion: *r* = −0.647, 95% CI = −0.8 to −0.5, *P* < 0.001, 41.9%; and bench press: *r* = −0.777, 95% CI = −0.9 to −0.6, *P* < 0.001, 60.4%). When only the masters athletes were included, high negative correlations were observed between age and CMJ and age and bench press (CMJ: *r* = −0.782, 95% CI = −0.9 to −0.6, *P* < 0.001; bench press: *r* = −0.776, 95% CI = −0.9 to −0.6, *P* < 0.001), with age accounting for 61.2% and 60.2% of the variation, respectively. Between age and knee flexion, only a modest negative correlation was detected (*r* = −0.469, 95% CI = −0.7 to −0.2, *P* = 0.002), with age accounting for 22% of the variation in knee flexion strength.

Among the body anthropometric variables, modest negative correlations were observed between age and height, age and weight, and age and LBM when all the athletes were included (height: *r* = −0.512, 95% CI = 0.7 to −0.3, *P* < 0.001; weight: *r* = −0.419, 95% CI = −0.6 to −0.2, *P* < 0.001; LBM: *r* = −0.525, 95% CI = −0.7 to −0.3, *P* < 0.001), with age accounting for 26.2%, 17.6%, and 27.6% of the variation, respectively. Of the blood cell counts, low to modest negative correlations were detected between age and lymphocyte percentage and age and RBC when all the athletes were included (LYM%: *r* = −0.384, 95% CI = −0.6 to −0.2, *P* = 0.002; RBC: *r* = −0.433, 95% CI = −0.6 to −0.2, *P* < 0.001), with age accounting for 14.7% and 18.7% of the variation, respectively. In addition, a modest positive correlation was detected between age and neutrophil percentage (*r* = 0.411, 95% CI = 0.2 to 0.6, *P* = 0.001), with age accounting for 16.9% of the variation in neutrophils. When only the masters athletes were included, the pattern was similar in slightly weaker age correlations (LYM%: *r* = −0.315, 95% CI = −0.6 to 0.0, *P* = 0.031; RBC: *r* = −0.370, 95% CI = −0.6 to −0.1, *P* = 0.010; and NEUT%: *r* = 0.326, 95% CI = 0.0 to 0.6, *P* = 0.025), with age accounting for 9.9%, 13.7%, and 10.6% of the variation, respectively. A low negative correlation was observed between age and HGB both when all the athletes were included (*r* = −0.383, 95% CI = −0.6 to −0.2, *P* = 0.002) and when only the masters athletes were included (*r* = −0.370, 95% CI = −0.6 to −0.1, *P* = 0.010), with age accounting for 14.7% and 13.7% of the variation in HGB, respectively. Of the studied serum molecules, a modest negative correlation between age and FasL was detected when all the athletes were included in the analyses (*r* = −0.596, 95% CI = −0.7 to −0.4, *P* < 0.001), with age accounting for 35.5% of the variation in FasL concentration. A modest positive correlation was detected between age and miR-146a when all the athletes were included (*r* = −0.611, 95% CI = −0.7 to −0.4, *P* < 0.001), with age accounting for 37.3% of the miR-146a variation. No significant correlations between age and the serum molecules were detected when only masters athletes were included in the analyses.

### 3.2. Changes in the Physical Performance of Masters Athletes over 10 Years

Changes in the training frequencies and the change percentage of the specific physical performance measures in 10 years are presented in Table 3. The results are shown for masters sprinters as a whole and divided into 3 age groups (B: 50–66 yrs, C: 66–79 yrs, and D: 79–90 yrs presenting the end-point ages). The self-reported weekly training frequency, sprint, and other training hours decreased when all the masters athletes were involved in the analyses (*P* < 0.001, *P* < 0.001, and *P* = 0.003, resp.). When grouping the athletes, the declines in all the self-reported training activities remained significant for the group B (*P* = 0.011, *P* = 0.009, and *P* = 0.008, resp.). For group C, no significant changes in the training activities over 10 years were reported. For the group D, weekly training frequency and sprint training hours decreased significantly (*P* = 0.004, *P* = 0.023, resp.). From the physical performance measures 60 m sprint, CMJ and isometric bench press changed during the 10 years' follow-up period when all the masters sprinters were included (*P* < 0.001 for all measures). When the masters sprinters were divided into 3 age groups the changes remained for all groups B (*P* = 0.001, *P* = 0.001, and *P* = 0.036, resp.), C (*P* = 0.002, *P* = 0.001, and *P* = 0.008, resp.), and D (*P* = 0.005, *P* < 0.001, and *P* < 0.001, resp.), with the highest deterioration seen among the oldest athletes (group D). There were no significant changes in the knee flexion strength in 10 years.

### 3.3. Changes in the Serum hsCRP, FasL, miR-21, and miR-146a Levels of Masters Athletes over 10 Years

The change percentages of the blood parameters, hsCRP, FasL, miR-21, and miR-146a over 10 years are presented in Table 3. The results are shown for the masters sprinters both as a whole and divided into 3 age groups (B: 50–66 yrs, C: 66–79 yrs, and D: 79–90 yrs presenting the end-point ages). Serum hsCRP did not change over the 10-year period among the athletes. The serum FasL concentrations decreased (*P* = 0.017) and serum miR-21 and miR-146a levels increased (*P* < 0.001, *P* < 0.005, resp.) among the masters sprinters as a single group during the 10 years. At the age-group level, the changes were significant for FasL and for miR-21 among the youngest masters sprinters (group B; *P* = 0.001*; P* = 0.007, resp.) and for miR-146a among the oldest group (D; *P* = 0.011). The change was also significant for miR-21 in the oldest group (group D, *P* = 0.017). The original values for the blood parameters are presented in supplementary data (S2).

### 3.4. FasL, miR-21, and miR-146a Associations with Physical Performance Measures in the 10-Year Follow-Up

**Figure 2:**
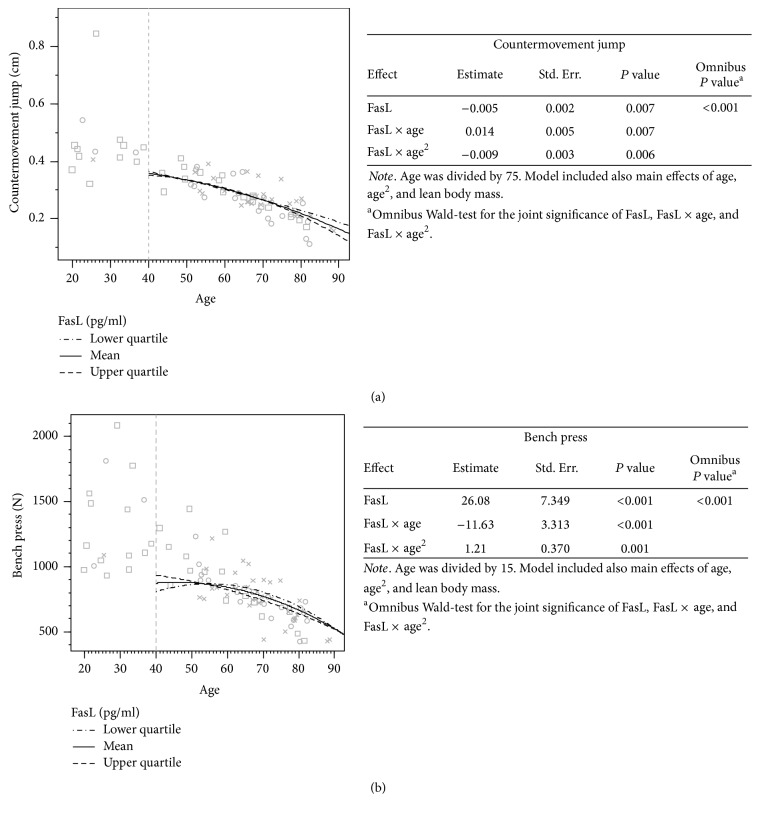
The association of serum FasL concentration with physical performance over time. Values for the younger participants (<40 years) without follow-up measures are presented on the left side of the images. The associations are based on the follow-up design (*n* = 49, >40 yrs). Cross (×) indicates that case is located within 0–37.5%, circle (○) within 37.5–67.5%, and square (□) within 67.5–100% of the cumulative share of the FasL distribution. The 3 different lines present the associations of the different serum marker levels with the physical performance measures over time. The tables next to the curves present the model used in forming the prediction curves, in greater detail, including statistics on the main effects of the studied serum marker and the possible quadratic and cubic effects.

**Figure 3:**
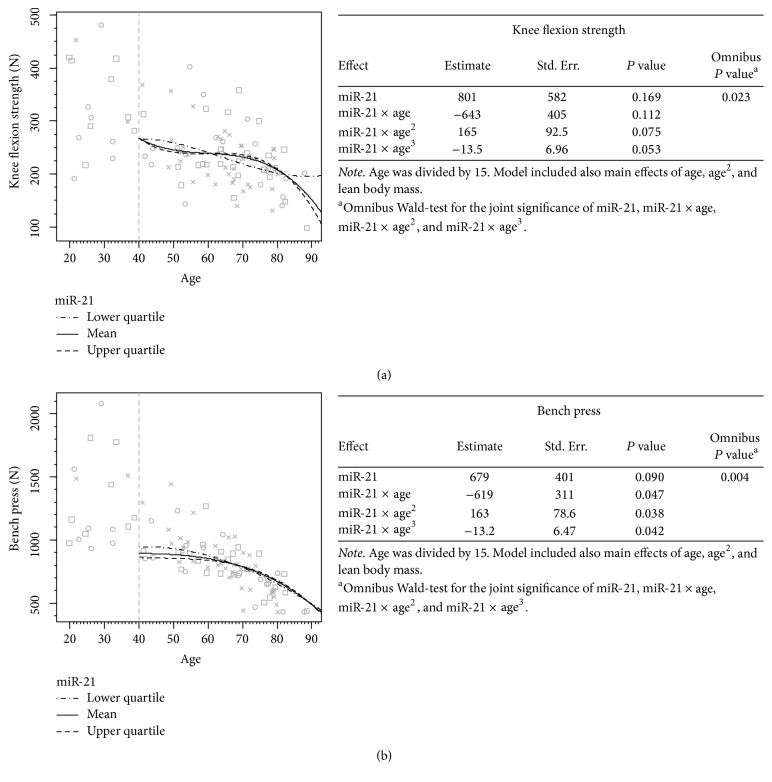
The association of serum miR-21 level with physical performance over time. Values for the younger participants (<40 yrs) without follow-up measures are presented on the left side of the images. The predictions are based on the follow-up design (*n* = 49, >40 yrs). Cross (×) indicates that case is located within 0–37.5%, circle (○) within 37.5–67.5%, and square (□) within 67.5–100% of the cumulative share of the miR-21 -distribution. The 3 different lines present the associations of the different serum marker levels with the physical performance measures over time. The tables next to the curves present the model used in forming the prediction curves, in greater detail, including statistics on the main effects of the studied serum marker and the possible quadratic and cubic effects.

**Figure 4:**
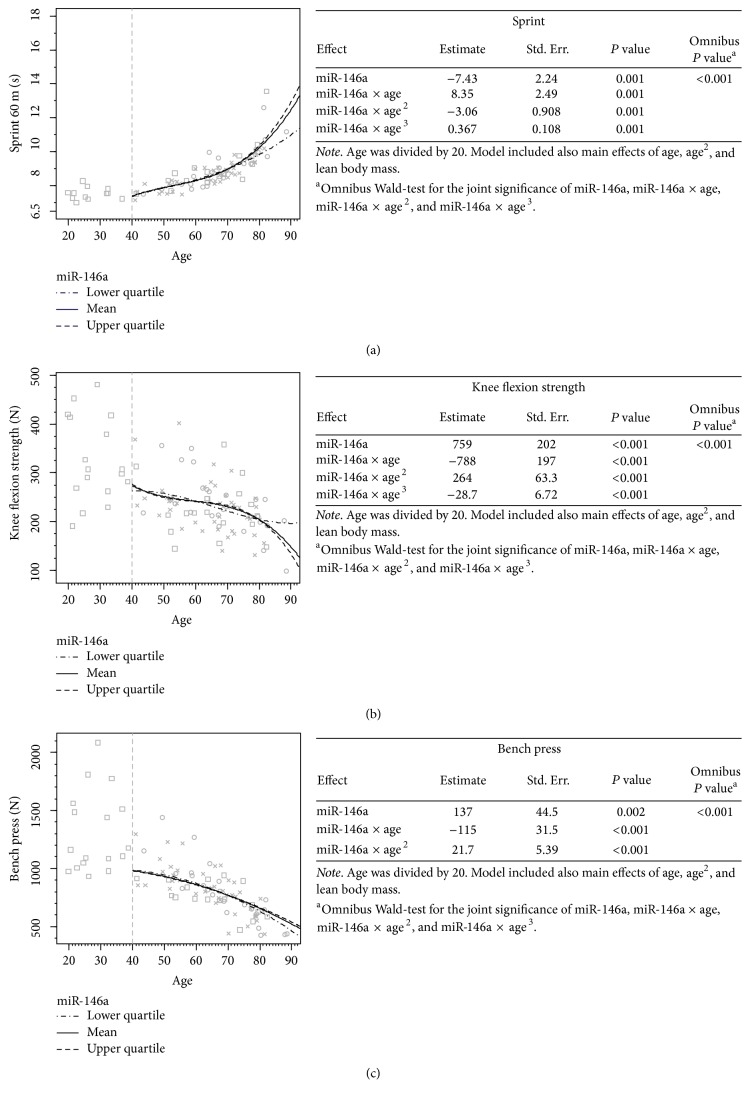
The association of serum miR-146a level with physical performance over time. Values for the younger participants (<40 years) without follow-up measures are presented on the left side of the images. The predictions are based on the follow-up design (*n* = 49, >40 yrs). Cross (×) indicates that case is located within 0–37.5%, circle (○) within 37.5–67.5%, and square (□) within 67.5–100% of the cumulative share of the miR-146a distribution. The 3 different lines present the associations of the different serum marker levels with the physical performance measures over time. The tables next to the curves present the model used in forming the prediction curves, in greater detail, including statistics on the main effects of the studied serum marker and the possible quadratic and cubic effects.

A GEE model was constructed to combine the effects of a specific serum molecule (FasL, miR-21, or miR-146a) and the physical performance measures (60 m sprint, CMJ, knee flexion, or bench press) over time. The association curves for the physical performance measures according to the different measured circulating molecule levels are presented in Figures 2, 3, and 4. Only significant or trending curves are presented. The results for the models were adjusted with the LBM.  Figure 2 shows that the combination of the effects of FasL and its interactions with age was statistically significant for the CMJ (*P* < 0.001) and bench press (*P* < 0.001). Also, all coefficient estimates for these outcomes were statistically significant indicating both a significant linear and quadratic curvature term. The model for the CMJ, when the serum FasL concentration was taken into account (Figure 2(a)), predicted a steeper decline in performance after age of 70 with slightly higher FasL serum concentrations than with lower FasL levels. For bench press, higher FasL levels at the younger ages predicted a steadier decline in performance while lower FasL values predicted better sustained performance at the older ages (Figure 2(b)).

Significant effects of miR-21 and age combined (Figure 3) were detected for knee flexion (*P* = 0.023) and bench press strength (*P* = 0.004). Statistically significant coefficient estimates relate mainly to miR-21 interaction with quadratic of cubic terms of age indicating that curvature has a stronger role in the prediction. For knee flexion strength, when serum miR-21 levels were taken into account, low miR-21 levels predicted best performance prior to age 60 and high levels best performance between ages 60 and 80 (Figure 3(a)). Low miR-21 levels predicted highest performance in the bench press until age 65, after which the prediction curves were very similar to each other (Figure 3(b)).

Significant combination effects of miR-146a and age (Figure 4) were detected for sprint (*P* < 0.001), knee flexion (*P* < 0.001), and bench press strength (*P* < 0.001). The miR-146a levels predicted the largest differences in 60 m sprint performance after the age of 70, after which the lowest values predicted the best sprint performance (Figure 4(a)). For knee flexion strength, the lowest miR-146a levels predicted the best performance until age 60, after which, until age 80, the highest serum levels predicted the best performance in the knee flexion strength (Figure 4(b)). For bench press strength, the lowest miR-146a values predicted the highest performance until age 65, after which the lowest miR-146a levels predicted the steepest decline (Figure 4(c)).

## 4. Discussion

This study investigated the associations of circulating levels of traditional (hsCRP, leukocyte count, and FasL) and novel (miR-21 and miR-146a) inflammation- and apoptosis-related molecules with physical performance in competitive male sprinters of different ages. In addition, the associations of serum FasL, miR-21, and miR-146a levels with specific physical performance measures and aging were determined. We used both cross-sectional and follow-up study designs, with an emphasis on the latter, which focused on older masters sprinters. In the cross-sectional analysis, which included sprinters from ages 18 to 90 years, anthropometrics, LBM, physical performance measures, RBC, and HGB were, as expected, negatively associated with aging. For the traditional inflammation markers, no age-association was observed with hsCRP; instead, the percentage of serum lymphocytes decreased and that of neutrophils increased, with age. Serum FasL concentration and miR-146a levels correlated negatively with age when all the sprinters were included. However, when only the masters sprinters were studied, the age correlation was not significant, indicating that the most radical changes in these molecules generally occur during the interval between being a young sprinter and becoming a masters sprinter. The 10-year follow-up study design, which concerned masters sprinters only, showed, as expected, a worsening of physical performance in parallel with the decrement in the serum FasL and increment in the serum miR-21 and miR-146a levels. Interestingly, when grouped into 3 different age groups we obtained novel information about the time frames of the changes. For FasL and miR-21 the changes were significant for the 50- to 66-year-old (end-point age) sprinters and for miR-21 and miR-146a for the 79- to 90-year-old sprinters. No significant changes were observed in the 66–79-year-old athletes. In addition, associations with serum molecules, physical performance and aging were determined. We found nonlinear associations of circulating FasL concentration with CMJ height and bench press strength. MiR-21 levels were associated with knee flexion and bench press strength and miR-146a levels with sprint time, knee flexion, and bench press strength. The associations were based on the 10-year-follow-up data and age was used as a continuous determinant. Based on the constructed model, it is possible to predict whether and how the different levels of the studied serum molecules explain the declining physical performance measures over time.

Aging is accompanied with declining skeletal muscle properties and increasing numbers of systemic classical inflammatory markers, which, in general, affect physical functioning [27]. Inflammation and apoptosis are two crucial processes known to be altered during the aging process having broad physiological or even pathological influences in the body [28, 29]. Prolonged physical training has been shown to improve the systemic inflammatory state, especially by lowering hsCRP and IL-6 levels, as well as preventing the loss of muscle mass [3]. In the present study, hsCRP neither differed between the studied age groups nor changed during the 10-year follow-up among the masters athletes. Therefore, we focused on the more specific circulating molecules, FasL, miR-21, and miR-146a, interplaying with aging, inflammation, apoptosis, and skeletal muscle tissue [6, 10–14].

### 4.1. FasL as a Potential Biomarker

Serum FasL contributes to cellular homeostasis by inducing apoptosis of the target cells, especially T lymphocytes [6]. Serum FasL concentrations have been shown to decrease with aging [29, 30], with higher serum FasL levels being associated with diseases related to imbalanced homeostasis of the immune cells [31–33]. As the follow-up results show, the most radical change in serum FasL levels in the present study had occurred by age 66, with the levels having decreased significantly by that age. This could be interpreted as a decrement in the apoptotic rate. Lower serum FasL level predicted better overall performances (CMJ, bench press). In light of both these studies and our findings, the natural decrement in FasL during aging could be beneficial for balancing the changing metabolism and inflammatory status. However, conflicting studies and theories exist. The shift towards reduced apoptosis, measured by decreased serum FasL levels, could be followed by an accumulation of immune cells, resulting in “inflammaging” or an accumulation of other cell types, thereby increasing the risk for cancer development (reviewed by Tower [34]). However, the traditional inflammation marker hsCRP levels of the oldest athletes in the present study would appear to be in the normal healthy range and show no indication of an increased inflammatory state. Instead, the measured higher count of circulating neutrophils among older sprinters could be an indication of slightly higher inflammatory status compared to younger athletes. However, the role of neutrophils as driving forces of tissue repair and regeneration has also recently been discussed (reviewed by Jones et al. [35]). Immune cell homeostasis has been shown to differ between men with opposite training background [7]. The authors showed that the basal level of lymphocyte apoptosis, controlled by Fas-FasL interaction, is distinctly different between high and low trained men, being higher among the former. However, right after a bout of acute exercise, the high-trained men seemed to be more resistant to exercise-induced apoptosis. It is possible that, through physiological adaptations induced by long-term training, the basal levels of serum FasL could be kept at low levels without adding to inflammatory status; instead the postexercise condition would function in its own, adapted, way. However, this notion needs to be addressed by a study that also includes nontrained sedentary people.

### 4.2. miR-21 as a Potential Biomarker

miRs are regulating several biological processes in cells including those associated with adaptation to exercise, inflammation, and apoptosis (see S3). miR-21 is widely known as an antiapoptotic-miR owing to its presence at high levels in several malignancies. Its systemic levels have also been shown to be upregulated in elderly people and its possible role as an inflammatory marker has been discussed [10]. In the present study, miR-21 levels increased significantly in the earlier years (40+), leveled out through the middle years (56+) and again increased significantly in the later years (69+). The self-reported training histories showed that the most significant decline in training occurred among the 40+ group and 69+ group, showing the opposite pattern to that of the miR-21 levels in those age groups. However, the associations between the change in sprint-specific training and the change in miR-21 levels (data not shown) were analyzed and no significant correlations were found (*R*^2^ = 0.074; *P* = 0.081), indicating that the decline in training did not explain the increments in serum miR-21. Our association analyses indicate that lower miR-21 levels are more beneficial for knee flexion and bench press performance. These findings support the idea that the higher the level of miR-21, the more unbeneficial it is for physiological status. Our results are also in line with the study by Wardle et al. [19], who reported that young male strength athletes have lower levels of plasma miR-21 than endurance athletes, which could be a beneficial result favoring strength training.

### 4.3. miR-146a as a Potential Biomarker

miR-146a has been proposed as an anti-inflammatory miR, negatively regulating the inflammatory response by targeting TNF receptor-associated factor 6 (TRAF-6) and IL-1R-associated kinase (IRAK-1) [11]. In the present study, miR-146a serum levels increased significantly among the oldest participants after age 69. This finding raises the question of whether circulating miR-146a is one of the regulators and a component of the training-induced adaptation system, needed to balance the inflammatory status in the elderly. In the sprint association, the miR-146a levels at the earlier ages did not seem to predict performance in the later years; however, with lower levels after age 70, better 60 m sprint time was obtained. In the knee flexion association, similar results were obtained for miR-21: with lower levels, better performance was obtained in the later years. In the bench press association, with higher miR-146a levels, slightly better results in bench press performance were obtained in the later years. These results for miR-146a and physical performance associations in aging showed a distinct pattern for the sprint versus bench press, with lower levels being more beneficial for sprint and higher levels for bench press in the later years.

Masters athletes demonstrate that, with an active, motivated, and healthy lifestyle, aging does not inevitably lead to physical frailty and disability [36]. In our study, even explosive strength and sprinting performances were preserved at relatively high levels into old age, even if some athletes reported a decline in their training activity over the 10-year follow-up. However, despite habitual training, after 80 years of age the hitherto modest decline in the performance assumes a more radical form. It has been suggested that in old age the curvilinear decline in physical performance may be explained by a concomitant deterioration in several physiological systems [37]. The role of circulating molecules delivering intercellular messages in these deteriorative events is evident, however, very complex. In the present study, the decline in the physical performance measures over time was partially explained by changes in the serum FasL, miR-21, and miR-146a levels, molecules associated with inflammation and cellular homeostasis. More detailed functional and tissue specific studies are thus needed to better understand the role and regulation of these potential biomarkers in aging and training adaptations.

## 5. Conclusions

The main focus of the study was to determine whether specific circulating inflammation- and apoptosis-related molecules, that is, FasL, miR-21, and miR-146a, are associated with physical performance and aging among masters sprinters. Previous studies have demonstrated distinct associations of the studied molecules with physical performance and with age, but longitudinal combined associations have not been reported. We showed that the systemic levels of these molecules change over 10-year period and that associations exist between the molecules, specific physical performance measures, and aging. In addition, the associations with physical performances were slightly different depending on the age of the masters sprinters. Lower levels of FasL and miR-21 seemed to have more beneficial association with the performance measures generally, whereas the associations between miR-146a and performance are more dependent on the specific type of physical performance measure used. Further research with well-controlled study designs and populations are needed to determine whether these molecules are useful as biomarkers in the prediction of successful aging or identification of individuals at high risk for deterioration in performance with older age. In addition, the origin of the circulating biomarkers remains to be clarified.

## Supplementary Material

Supplementary material provides more detailed information about the prediction equations derived from the GEE model parameters. The models were used as a tool for estimating the body lean-adjusted associations of serum molecules with physical performance measures over age.

## Figures and Tables

**Table 1:** Self-reported training history and physical performance measures in different age groups and correlations of the variables with age.

	A 18–39 yrs (*n* = 18)	B 50–66 yrs (*n* = 16)	C 66–79 yrs (*n* = 18)	D 79–90 yrs (*n* = 15)	Correlation with age (all groups)/*P* value	95% CI/coefficient of determination	Correlation with age (only B, C, D)/*P* value	95% CI/coefficient of determination
*Self-reported training*								
Frequency (times/wk)	6.8 ± 2.3 (*n* = 15)	3.6 ± 1.6 (*n* = 14)	3.5 ± 1.3	3.1 ± 1.1 (*n* = 14)	−0.659 (*n* = 61) **P** < 0.001	−0.8 to −0.5 0.434	−0.176 (*n* = 46) *P* = 0.241	−0.4 to 0.1 0.031
Sprint training (h/wk)	7.0 ± 5.5	2.6 ± 1.7 (*n* = 14)	3.2 ± 3.2	2.7 ± 2.3 (*n* = 14)	−0.380^S^(*n* = 64) **P** = 0.002	−0.6 to −0.1 0.144	−0.085^S^(*n* = 46) *P* = 0.573	−0.3 to 0.2 0.007
Other training (h/wk)	2.5 ± 4.2	0.4 ± 0.6 (*n* = 14)	1.3 ± 2.0	2.0 ± 2.5 (*n* = 14)	0.179^S^(*n* = 64) *P* = 0.157	−0.1 to 0.4 0.032	0.310^S^(*n* = 46) **P** = 0.036	0.0 to 0.6 0.096
*Physical performance*								
Sprint 60 m (s)	7.52 ± 0.36 (*n* = 12)	8.43 ± 0.63 (*n* = 13)	9.29 ± 0.61 (*n* = 13)	10.81 ± 1.30 (*n* = 10)	0.898^S^(*n* = 48) **P** < 0.001	0.8 to 0.9 0.806	0.841^S^(*n* = 36) **P** < 0.001	0.7 to 0.9 0.707
CMJ (cm)	45.7 ± 11.8 (*n* = 15)	32.0 ± 4.2 (*n* = 12)	25.8 ± 5.0 (*n* = 12)	18.9 ± 4.7 (*n* = 11)	−0.810 (*n* = 50) **P** < 0.001	−0.9 to −0.7 0.656	−0.782 (*n* = 35) **P** < 0.001	−0.9 to −0.6 0.612
Isometric knee flexion (N)	326 ± 86 (*n* = 17)	253 ± 57 (*n* = 15)	227 ± 34 (*n* = 14)	190 ± 49 (*n* = 12)	−0.647 (*n* = 58) **P** < 0.001	−0.8 to −0.5 0.419	−0.469 (*n* = 41) **P** = 0.002	−0.7 to −0.2 0.220
Isometric bench press (N)	1307 ± 347 (*n* = 17)	945 ± 159 (*n* = 14)	717 ± 175 (*n* = 13)	565 ± 126 (*n* = 11)	−0.777 (*n* = 55) **P** < 0.001	−0.9 to −0.6 0.604	−0.776 (*n* = 38) **P** < 0.001	−0.9 to −0.6 0.602

Table is formed based on the cross-sectional study design (2012) including all the athletes from ages 18 to 90 yrs. Results are presented as means ± SD. Age correlations are presented: (1) all athletes and (2) masters athletes only. CMJ: countermovement jump. ^S^Spearman's correlation coefficient.

**Table 2:** Participant characteristics and blood parameters in different age groups and correlations of the variables with age.

	A 18–39 yrs (*n* = 18)	B 50–66 yrs (*n* = 16)	C 66–79 yrs (*n* = 18)	D 79–90 yrs (*n* = 15)	Correlation with age (all groups)/*P* value	95% CI/coefficient of determination	Correlation with age (only B, C, D)/*P* value	95% CI/coefficient of determination
*Anthropometrics and body composition*								
Height (cm)	180.1 ± 5.0	178.2 ± 8.1	172.8 ± 4.7	172.4 ± 4.9	−0.512 (*n* = 67) **P** < 0.001	−0.7 to −0.3 0.262	−0.464 (*n* = 49) **P** = 0.001	−0.7 to −0.2 0.215
Weight (kg)	78.3 ± 6.8	80.2 ± 9.7	71.0 ± 6.2	69.4 ± 6.4	−0.419 (*n* = 67) **P** < 0.001	−0.6 to −0.2 0.176	−0.568 (*n* = 49) **P** < 0.001	−0.7 to −0.3 0.323
LBM (kg)	67.2 ± 5.7	66.3 ± 7.0	60.2 ± 4.1	58.3 ± 4.8	−0.525 (*n* = 67) **P** < 0.001	−0.7 to −0.3 0.276	−0.564 (*n* = 49) **P** < 0.001	−0.7 to −0.3 0.318
Body fat mass (kg)	11.2 ± 4.1	13.9 ± 6.1	11.1 ± 3.7	11.2 ± 3.6	−0.028 (*n* = 67) *P* = 0.857	−0.3 to 0.2 0.001	−0.301 (*n* = 49) **P** = 0.035	−0.5 to 0.0 0.091
*Blood cell count*								
WBC	5.6 ± 1.4	5.6 ± 1.0	5.7 ± 2.2 (*n* = 17)	5.4 ± 0.8 (*n* = 14)	−0.057 (*n* = 65) *P* = 0.652	−0.3 to 0.2 0.003	0.099 (*n* = 47) *P* = 0.509	−0.2 to 0.4 0.010
LYM%	39.2 ± 7.2	35.9 ± 6.2	34.2 ± 5.6 (*n* = 17)	31.0 ± 9.4 (*n* = 14)	−0.384 (*n* = 65) **P** = 0.002	−0.6 to −0.2 0.147	−0.315 (*n* = 47) **P** = 0.031	−0.6 to 0.0 0.099
MXD%	12.3 ± 4.8	11.5 ± 3.0	11.5 ± 1.9 (*n* = 17)	10.9 ± 2.4 (*n* = 14)	−0.174 (*n* = 65) *P* = 0.165	−0.4 to 0.1 0.030	−0.112 (*n* = 47) *P* = 0.455	−0.4 to 0.2 0.013
NEUT%	48.6 ± 8.5	52.6 ± 6.9	54.3 ± 6.6 (*n* = 17)	58.1 ± 9.5 (*n* = 14)	0.411 (*n* = 65) **P** = 0.001	0.2 to 0.6 0.169	0.326 (*n* = 47) **P** = 0.025	0.0 to 0.6 0.106
RBC	5.1 ± 0.4	4.9 ± 0.4	4.9 ± 0.4 (*n* = 17)	4.4 ± 0.3 (*n* = 14)	−0.443 (*n* = 65) **P** < 0.000	−0.6 to −0.2 0.187	−0.370 (*n* = 47) **P** = 0.010	−0.6 to −0.1 0.137
PLT	211.9 ± 44.0	242.3 ± 44.3	210.0 ± 83.6 (*n* = 17)	199.7 ± 42.9 (*n* = 14)	−0.100 (*n* = 65) *P* = 0.429	−0.3 to 0.1 0.010	−0.375 (*n* = 47) **P** = 0.009	−0.6 to −0.1 0.141
HGB	154.3 ± 9.3	152.9 ± 12.4	149.4 ± 9.3 (*n* = 17)	140.3 ± 8.0 (*n* = 14)	−0.383 (*n* = 65) **P** = 0.002	−0.6 to −0.2 0.147	−0.443 (*n* = 47) **P** = 0.002	−0.6 to −0.2 0.187
*Serum molecules*								
hsCRP (mg/L)	1.6 ± 5.1	1.7 ± 3.3	2.3 ± 6.0 (*n* = 17)	1.3 ± 1.2 (*n* = 14)	−0.035 (*n* = 65) 0.784	−0.3 to 0.2 0.001	−0.102 (*n* = 47) *P* = 0.999	−0.4 to 0.2 0.010
FasL (pg/ml)	92.3 ± 24.7	56.8 ± 21.9 (*n* = 14)	56.0 ± 18.2 (*n* = 14)	52.3 ± 23.4 (*n* = 13)	−0.596 (*n* = 59) **P** < 0.001	−0.7 to −0.4 0.355	−0.181 (*n* = 41) *P* = 0.258	−0.5 to 0.1 0.033
miR-21 (RE)	1.81 ± 0.75	2.22 ± 1.41	1.74 ± 1.05	1.65 ± 0.62	−0.095^S^ (*n* = 67) *P* = 0.444	−0.3 to 0.1 0.009	−0.118^S^ (*n* = 49) *P* = 0.419	−0.4 to 0.2 0.014
miR-146a (RE)	5.82 ± 2.66	1.83 ± 1.16	1.50 ± 0.71	1.51 ± 0.96	−0.611^S^ (*n* = 67) **P** < 0.001	−0.7 to −0.4 0.373	−0.105^S^ (*n* = 49) *P* = 0.473	−0.4 to 0.2 0.011

Table is formed based on the cross-sectional study design (2012) including all the athletes from ages 18 to 90 yrs. Results are presented as means ± SD. Age correlations are presented in two ways: (1) one including all the athletes and (2) one including only masters athletes. LBM: lean body mass, WBC: white blood cells, LYM: lymphocytes, MXD: mixed leukocytes, NEUT: neutrophils, RBC: red blood cells, PLT: platelet, HGB: hemoglobin, hsCRP: high sensitivity c-reactive protein, FasL: Fas-ligand, and RE: relative expression. ^S^Spearman's correlation coefficient.

**Table 3:** The change in physical performance measures, self-reported training amounts, and serum molecule levels among all and age-grouped masters sprinters in the 10-year follow-up.

	All 50–90 yrs	B 50–66 yrs	C 66–79 yrs	D 79–90 yrs
*Training frequency *				
Change (times/wk)	−0.89 ± 1.22 (*n* = 42)	−0.91 ± 1.15 (*n* = 14)	−0.73 ± 1.43 (*n* = 15)	−1.06 ± 1.09 (*n* = 13)
*P* value	**P < 0.001**	**P = 0.011**	*P* = 0.067	**P = 0.004**
*Sprint training *				
Change (h/wk)	−1.84 ± 3.00 (*n* = 42)	−1.80 ± 2.25 (*n* = 14)	−1.94 ± 4.08 (*n* = 15)	−1.77 ± 2.43 (*n* = 13)
*P* values	**P < 0.001**	**P = 0.009**	*P* = 0.078	**P = 0.023**
*Other training*				
Change (h/wk)	−1.02 ± 2.62 (*n* = 32)	−1.53 ± 1.61 (*n* = 11)	−0.93 ± 3.44 (*n* = 11)	−0.56 ± 2.49 (*n* = 10)
*P* value	**P = 0.006**	**P = 0.008**	*P* = 0.221	*P = 0.424*

*Sprint 60 m (s)*				
Change%	11.9 (6.7−16.5) (*n* = 35)	8.2 (5.7−10.0) (*n* = 13)	12.2 (5.7−16.4) (*n* = 12)	15.8 (12.1−22.8) (*n* = 10)
*P* value	**P < **0.001^W^	**P = **0.001^W^	**P = **0.002^W^	**P = **0.005^W^
*CMJ (cm)*				
Change%	−15.4 ± 10.2 (*n* = 32)	−9.2 ± 6.3 (*n* = 11)	−15.7 ± 10.0 (*n* = 11)	−22.0 ± 10.4 (*n* = 10)
*P* value	**P < 0.001**	**P = 0.001**	**P = 0.001**	**P < 0.001**
*Knee flexion (N)*				
Change%	−1.8 ± 23.6 (*n* = 40)	5.5 ± 29.0 (*n* = 15)	−3.6 ± 20.1 (*n* = 13)	−9.07 ± 18.0 (*n* = 12)
*P* value	*P* = 0.062	*P* = 0.896	*P* = 0.287	*P* = 0.111
*Isometric bench press (N)*				
Change%	−10.9 ± 11.9 (*n* = 36)	−5.3 ± 9.8 (*n* = 14)	−14.0 ± 14.7 (*n* = 12)	−14.9 ± 8.51 (*n* = 10)
*P* value	**P < 0.001**	**P = 0.036**	**P = 0.008**	**P < 0.001**

*hsCRP*				
Change%	112 ± 349 (*n* = 47)	203 ± 512 (*n* = 16)	71.5 ± 230 (*n* = 17)	56.8 ± 220 (*n* = 14)
*P* value	*P* = 0.841	*P* = 0.404	*P* = 0.868	*P* = 0.220
*FasL*				
Change%	−5.3 ± 26.3 (*n* = 41)	−18.3 ± 16.7 (*n* = 14)	1.22 ± 29.1 (*n* = 14)	1.78 ± 27.9 (*n* = 13)
*P* value	**P = 0.017**	**P = 0.001**	*P* = 0.746	*P* = 0.587
*miR-21*				
Change%	77.0 (1.1–689) (*n* = 49)	107 (21.0–662) (*n* = 16)	37.9 (−34.7–834) (*n* = 18)	136 (21.3–995) (*n* = 15)
*P* value	**P < **0.001^W^	**P = **0.007^W^	*P* = 0.267^W^	**P = **0.017^W^
*miR-146a*				
Change%	95.4 (−7.4–631) (*n* = 49)	93.0 (−3.0–1101) (*n* = 16)	18.6 (−26.0–444) (*n* = 18)	143 (53.6–861) (*n* = 15)
*P* value	**P = **0.005^W^	*P* = 0.079^W^	*P* = 0.500^W^	**P = **0.011^W^

The age ranges of the groups (B, C, and D) represent the ages at follow-up. Data are presented as means ± SD for parametric variables and as median (IQR) for nonparametric variables. ^W^*P* value was calculated with paired *t*-test for normally distributed variables and with Wilcoxon signed rank test for nonparametric variables. ^W^Wilcoxon signed rank test for nonparametric variables. CMJ = countermovement jump, hsCRP = high sensitivity C-reactive protein, and FasL: Fas-ligand.

## Additional Points

*Limitations and Benefits.* This study only concerned relatively healthy males with a regular training background. However, it has been emphasized that athletes themselves are the best controls in the study of inherent aging [37]. Small percentage of the masters sprinters, who had taken part in the baseline measurements in 2002, was not available for the study follow-up in 2012. It is likely, therefore, that the follow-up setting is based on the fittest sprinters; this should be considered when interpreting the results. It should also be noted that the greatest emphasis in the physical performance association curves is on the middle age range, where the number of the participants was the greatest. The predictions are the most reliable until the age of 85 years from which onwards the thinning of the subjects might have unwanted effects on the predictions. It is important to bear in mind that miRs are sensitive and easily affected by external and internal stimuli. It was not possible to control for all the potentially confounding stimuli that could have affected the circulating miR levels (such as personal diet, lack of sleep, and nondiagnosed conditions). Previous studies have reported that miR-146a reacts acutely to exercise [17, 38]. However, the present study population represents relatively healthy, regularly training males with a long training history. With their bodies being well adapted to even rather heavy exercise, no surprising acute effects on blood parameters during the brief physical measurements on the previous day of blood sampling were expected. The participants had a similar background in their training and other living habits (no regular smokers, no heavy drinking). Also, the fact that the study design was similar in both years strengthens longitudinal comparability. When studying athletes, the motivation to reach one's personal peak in measurements of performance is likely to be higher than in nonathletes. One of the major strengths of the study is the 10-year follow-up design: it is unique and leaves no space for genetic variation, which is an issue in cross-sectional designs, especially when studying partly genetically regulated biomarkers, as shown in our previous study with monozygotic twins [26].
