# Supplementary material for: Declining Physical Performance Associates with Serum FasL, miR-21, and miR-146a in Aging Sprinters
Source: Biomed Res Int. 2017 Jan 3;2017:8468469. doi: 10.1155/2017/8468469 (PMC5239835; doi:10.1155/2017/8468469)
Supplement: Supplementary file 1 — Supplementary material provides more detailed information about the prediction equations derived from the GEE model parameters. The models were used as a tool for estimating the body lean-adjusted associations of serum molecules with physical performance measures over age. [file 8468469.f1.pdf]

Supplementary data

S1: Prediction equations based on GEE-models

FasL

$$CMJ = 0.51 - 0.73\times\frac{Age}{75} + 0.31\times\left(\frac{Age}{75}\right)^2 - 0.0054\times FasL + 0.014\times FasL\times\frac{Age}{75} - 0.0090\times\left(\frac{Age}{75}\right)^2 + 0.0028\times BL$$

$$BP = -1127 + 934\times\frac{Age}{15} - 112\times\left(\frac{Age}{15}\right)^2 + 26\times FasL - 12\times FasL\times\frac{Age}{15} + 1.21\times\left(\frac{Age}{15}\right)^2 + 1.43\times BL$$

miR-21

$$KFS = -494 + 584\times\frac{Age}{15} - 152\times\left(\frac{Age}{15}\right)^2 + 12.2\times\left(\frac{Age}{15}\right)^3 + 801\times FasL - 643\times FasL\times\frac{Age}{15} + 165\times\left(\frac{Age}{15}\right)^2$$
$$-13\times\left(\frac{Age}{15}\right)^3 + 0.89\times BL$$

$$BP = -1802 + 147\times\frac{Age}{15} - 2.45\times\left(\frac{Age}{15}\right)^2 + 0.12\times\left(\frac{Age}{15}\right)^3 + 1092\times FasL - 63.6\times FasL\times\frac{Age}{15} + 1.12\times\left(\frac{Age}{15}\right)^2$$
$$-0.0062\times\left(\frac{Age}{15}\right)^3 - 0.018\times BL$$

miR-146a

$$S60 = 9.54 - 2.71\times\frac{Age}{20} + 1.15\times\left(\frac{Age}{20}\right)^2 - 0.11\times\left(\frac{Age}{20}\right)^3 - 7.43\times FasL + 8.35\times FasL\times\frac{Age}{20} - 3.06\times\left(\frac{Age}{20}\right)^2$$
$$+0.37\times\left(\frac{Age}{20}\right)^3 - 0.0063\times BL$$

$$KFS = -460 + 748\times\frac{Age}{20} - 255\times\left(\frac{Age}{20}\right)^2 + 26.9\times\left(\frac{Age}{20}\right)^3 + 759\times FasL - 788\times FasL\times\frac{Age}{20} + 264\times\left(\frac{Age}{20}\right)^2$$
$$-28.7\times\left(\frac{Age}{20}\right)^3 - 0.35\times BL$$

$$BP = 677 + 276\times\frac{Age}{20} - 77.2\times\left(\frac{Age}{20}\right)^2 + 137\times FasL - 115\times FasL\times\frac{Age}{20} + 21.7\times\left(\frac{Age}{20}\right)^2 + 1.11\times BL$$

Note. CMJ = countermovement jump, BP = bench press, KFS = knee flexion strength, S60 = Sprint (60 m), BL = bodylean. Age-scaling is necessary to bring variation in variables closer together, which enables convergence of the estimation process.

Table S1. Sample quantities for GEE-model predictors.

		Quartile	
	**Mean**	**Lower**	**Upper**
miR-21	1.55	0.75	2.07
miR-146a	0.61	1.58	1.88
FasL	59	41	76
Bodylean	62	59	66

S2: The table presents the values for the blood parameters from the same athletes in 2002 and 2012. Data are shown for the masters athletes as a single group (All;50-90 yrs in 2012) and as three age groups (B; 50-66 yrs, C: 66-79 yrs and D: 79-90 yrs in 2012)

	**2002**	**2012**	**P-value for the change**
**hsCRP (mg/L)**			
All (n=47)	2.0±4.2	1.8±4.1	0.841
B (n=16)	1.0±1.0	1.7±0.6	0.404
C (n=17)	2.6±0.8	2.3±0.7	0.868
D (n=14)	2.2±2.6	1.3±1.2	0.220
**FasL (pg/mL)**			
All (n=41)	60.7±24.8	55.1±20.8	0.017
B (n=14)	70.0±26.8	56.8±21.9	0.001
C (n=14)	57.3±19.5	56.8±18.2	0.746
D (n=13)	54.2±29.5	52.3±23.4	0.587
**miR-21 (RE)**			
All (n=49)	0.80 (0.29-1.29)	1.54 (1.11-2.25)	< 0.001[W]
B (=16)	0.76 (0.40-1.20)	1.64 (1.30-3.49)	0.007[W]
C (n=18)	0.97 (0.29-1.59)	1.37 (0.91-2.20)	0.267[W]
D (n=15)	0.78 (0.14-1.35)	1.50 (1.06-2.05)	0.017[W]
**miR-146a (RE)**			
All (n=49)	0.75 (0.23-1.25)	1.40 (0.87-2.13)	0.005[W]
B (=16)	0.68 (0.09-1.58)	1.65 (0.82-2.59)	0.079[W]
C (n=18)	0.97 (0.32-1.88)	1.31 (0.85-2.22)	0.500[W]
D (n=15)	0.62 (0.14-0.81)	1.34 (0.92-1.57)	0.011[W]

For the parametric variables, the results are presented as mean±SD and for non-parametric variables as median (IQR). Mann Whitney test was performed for parametric variables and Wilcoxon signed rank test for non-parametric variables. RE= relative expression

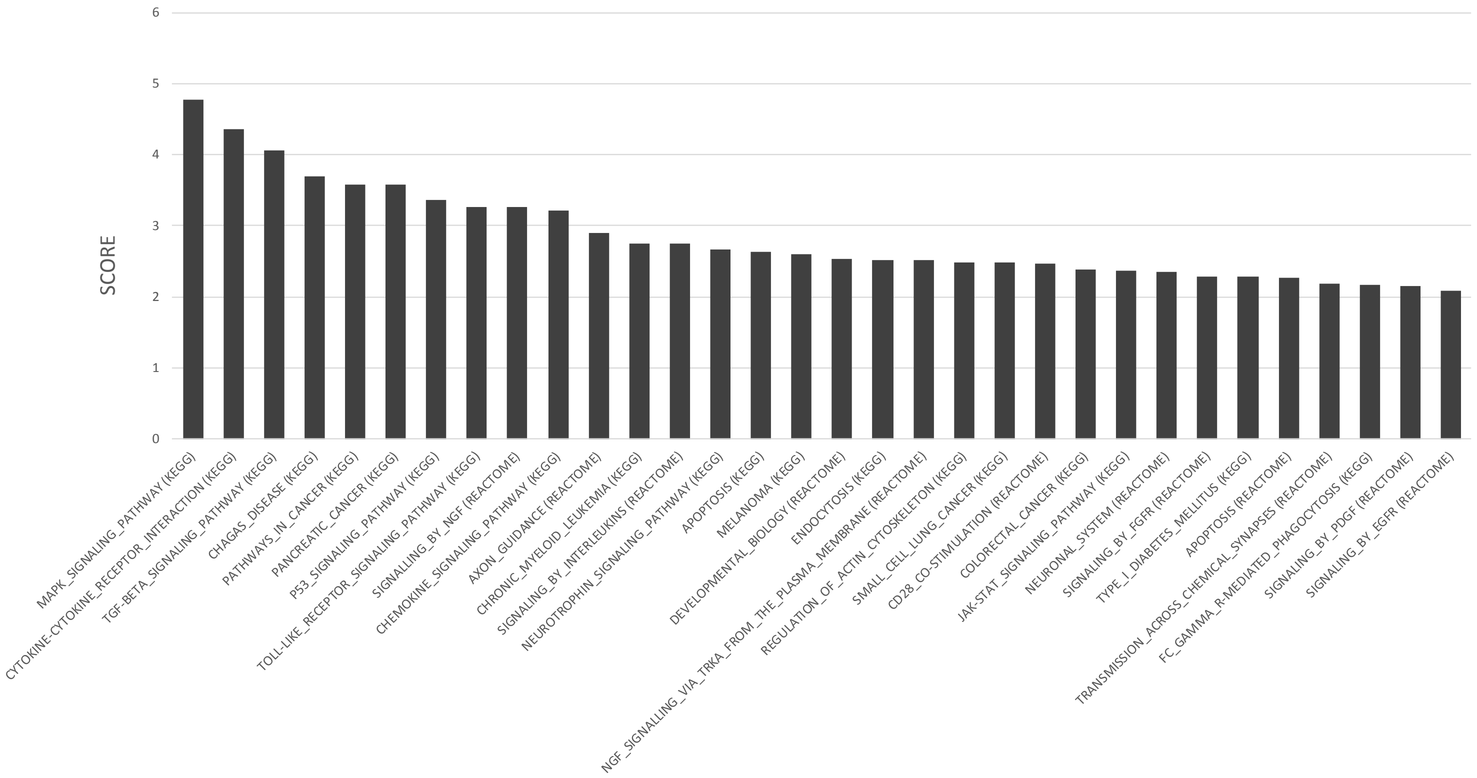

S3: Pathway analysis by miRsystem. Common pathways for miR-21 and miR-146a targets. Scores higher than 2 are presented in the figure. MirSystem version 20150312 was used for the analysis.
